# Expanding the horizon of CAR T cell therapy: from cancer treatment to autoimmune diseases and beyond

**DOI:** 10.3389/fimmu.2025.1544532

**Published:** 2025-02-19

**Authors:** Zishan Yang, Bingjun Ha, Qinhan Wu, Feng Ren, Zhinan Yin, Hongru Zhang

**Affiliations:** ^1^ Xinxiang Key Laboratory of Tumor Vaccine and Immunotherapy, School of Basic Medical Sciences, Xinxiang Medical University, Xinxiang, Henan, China; ^2^ Henan International Joint Laboratory of Immunity and Targeted Therapy for Liver-Intestinal Tumors, Xinxiang Medical University, Xinxiang, Henan, China; ^3^ State Key Laboratory of Medicinal Chemical Biology, Tianjin Key Laboratory of Protein Sciences, Cancer Biology Center, College of Life Sciences, Nankai University, Tianjin, China; ^4^ Zhuhai Precision Medical Center, Zhuhai People’s Hospital (Zhuhai Hospital Affiliated with Jinan University), Jinan University, Zhuhai, Guangdong, China; ^5^ The Biomedical Translational Research Institute, Jinan University, Guangzhou, Guangdong, China; ^6^ Nankai International Advanced Research Institute (Shenzhen Futian), Nankai University, Shenzhen, Guangdong, China

**Keywords:** CAR-T cells, adoptive immunotherapy, tumor microenvironment, solid tumors, autoimmune diseases

## Abstract

Chimeric antigen receptor (CAR)-T-cell therapy has garnered significant attention for its transformative impact on the treatment of hematologic malignancies such as leukemia and lymphoma. Despite its remarkable success, challenges such as resistance, limited efficacy in solid tumors, and adverse side effects remain prominent. This review consolidates recent advancements in CAR-T-cell therapy and explores innovative engineering techniques and strategies to overcome the immunosuppressive tumor microenvironment (TME). We also discuss emerging applications beyond cancer, including autoimmune diseases and chronic infections. Future perspectives highlight the development of more potent CAR-T cells with increased specificity and persistence and reduced toxicity, providing a roadmap for next-generation immunotherapies.

## 1 Introduction

Adoptive cell therapy (ACT) is a novel strategy for cancer treatment. Human T cells are modified *in vitro* with genetic engineering technology and then reinfused into patients for therapeutic purposes. In particular, T cells engineered to express chimeric antigen receptor (CAR) have been shown to have significant clinical success, and the prognoses of many patients with hematological diseases have improved (1–3). Anti-CD19 CAR-T cells have shown unprecedented clinical effects in treating various diseases, including acute lymphoblastic leukemia (ALL) in children and adults, non-Hodgkin lymphoma (NHL), diffuse large B-cell lymphoma (DLBCL), and chronic lymphocytic leukemia (CLL) (4–8).

**Figure 1:**
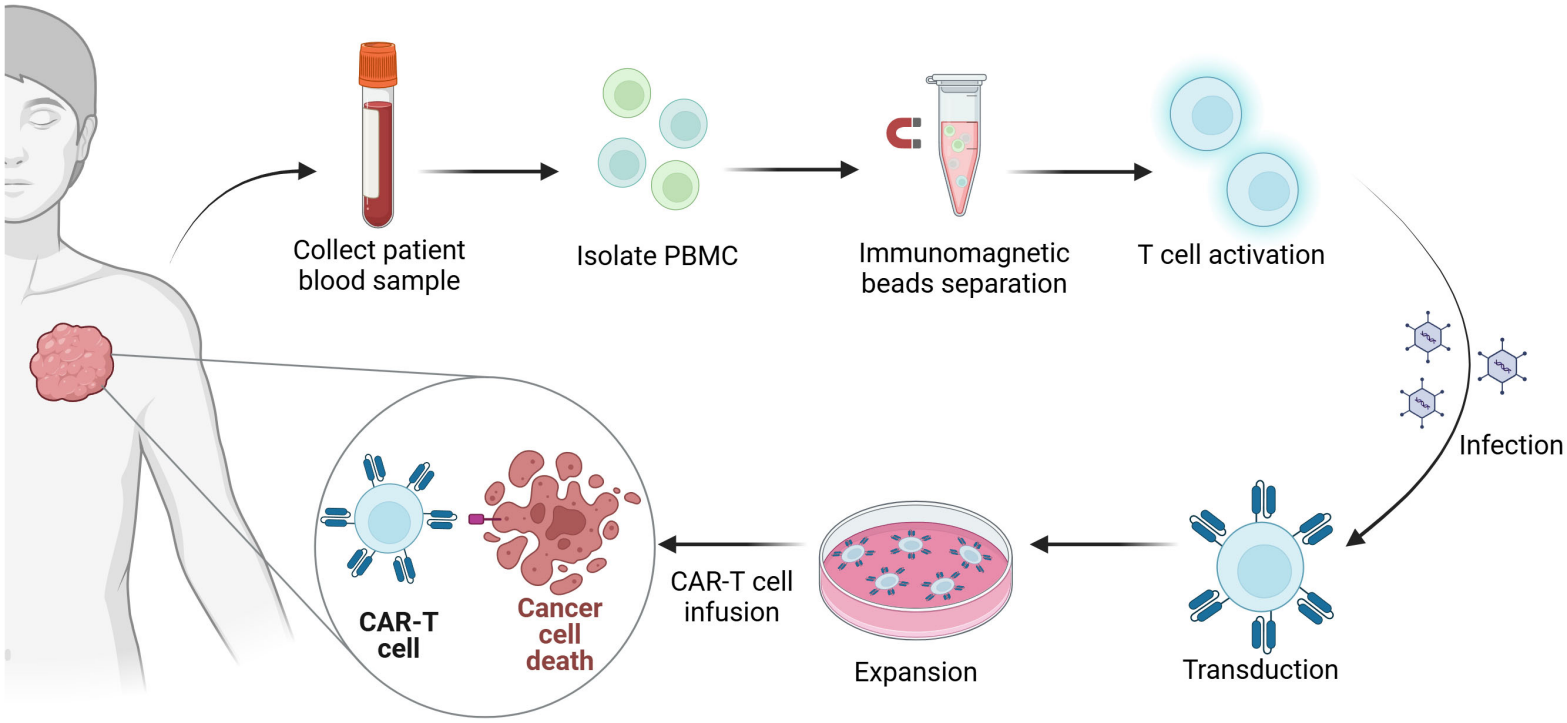
Manufacturing process of CAR-T-cell therapy. The first step in the generation of CAR-T cells is the collection of blood samples from the patient. Subsequently, peripheral blood mononuclear cells (PBMCs) are isolated from the collected blood samples. Immunomagnetic beads are utilized to isolate T cells, which are activated simultaneously. Next, the gene encoding the chimeric antigen receptor (CAR) is introduced into the T cells via viral infection. Finally, the CAR-T cells are expanded *in vitro* and then reinfused into the patient’s body to eradicate tumors.

At present, CAR-T cells are cultivated from T cells derived from peripheral blood *in vitro* and then genetically modified in the laboratory to introduce specific genes encoding CAR proteins so that these specific antigen receptors can be expressed on the T-cell surface. These engineered T cells are subsequently amplified in the laboratory and infused into the patient. Once in the body, they can identify and eliminate tumor cells bearing target antigens (**Figure 1**).

**Figure 2:**
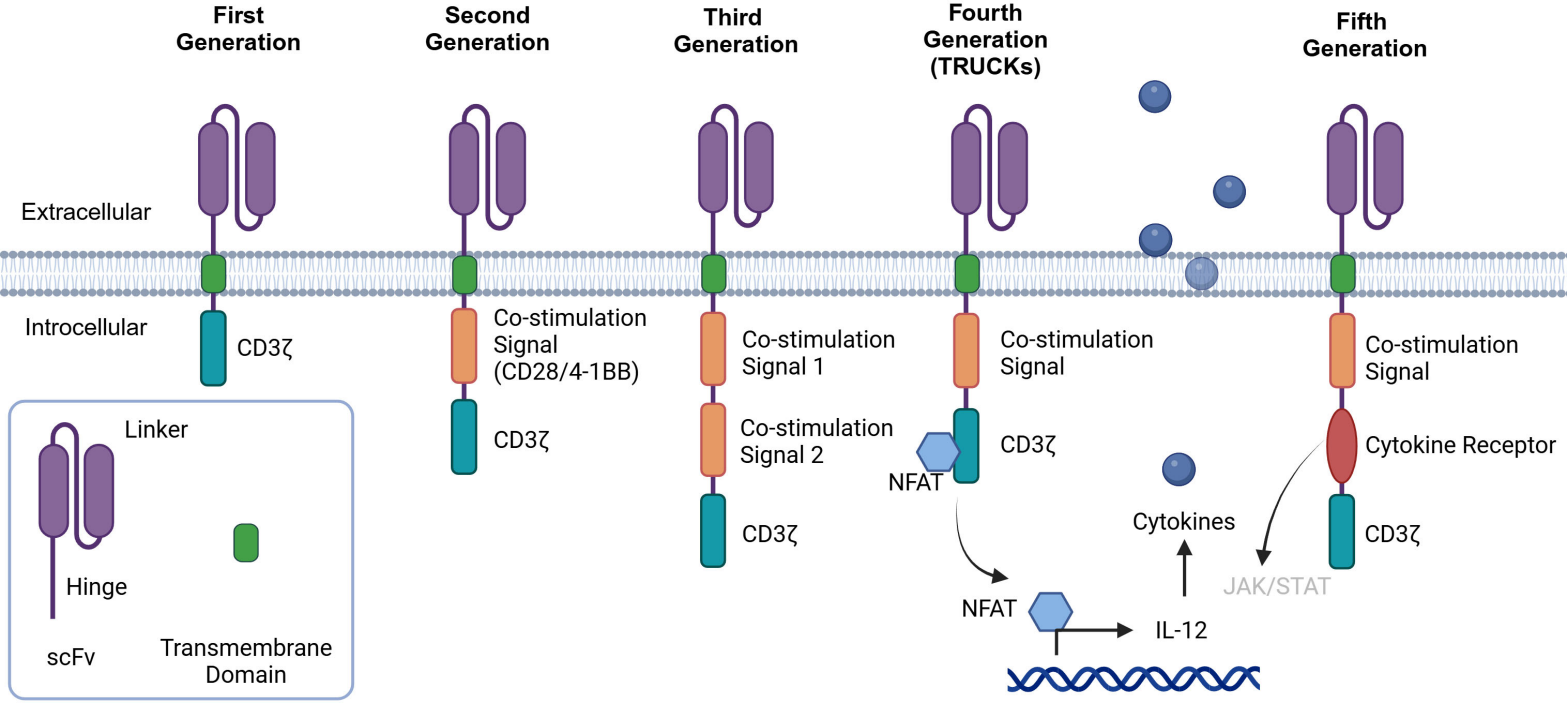
History of CAR-T-cell development. First-generation CAR-T cells are built upon the CD3-ζ chain. While they possess the ability to activate T cells, their antitumor efficacy is relatively limited. Second-generation CARs include a costimulatory molecule, such as CD28 or 4-1BB, which enhances the activation and functionality of CAR-T cells. Third-generation CARs further enhance the intracellular signaling domain by including a second costimulatory molecule. When a single-chain variable fragment (scFv) binds to tumor-associated antigens (TAAs), it activates the first signal through CD3ϵ and the second signal via two costimulatory signals, thereby strengthening the T-cell response. Fourth-generation CAR-T cells, also referred to as “cytokine-mediated killers at the cosmic level,” are engineered to release modified genes into tumor tissue upon the binding of the CAR to targeted antigens. This unique design aims to increase antitumor activity in a more targeted and potent manner. Fifth-generation CAR-T cells are designed by adding a cytoplasmic IL-2R β-chain domain and a STAT3/5 binding site to the second-generation design. This modification is expected to further optimize the activation, proliferation, and antitumor capabilities of CAR-T cells.

CAR is the key component of CAR-T cells responsible for binding to the corresponding antigens on the tumor cell surface for targeted therapy. Different tumor-associated antigens (TAAs) can be used as target antigens because of their specific high expression in tumor tissue. CAR has four major structures: an extracellular antigen recognition domain (usually a single chain variable fragment (scFv)), a hinge domain, a transmembrane domain, and an intracellular signal transduction domain (usually CD3ζ) (9, 10) (**Figure 2**). CAR is a synthetic receptor that enables T cells to recognize TAAs without the major histocompatibility complex (MHC). To improve the curative effects, five generations of CARs have been developed (**Figure 2**) (11). The first generation of CARs consisted of an extracellular antigen recognition domain fused with a transmembrane domain and the CD3ζ intracellular signaling domain (12). However, the results of clinical trials did not meet expectations because of the slow amplification and poor durability of the T cells. The second and third generations included one or two costimulating domains (usually CD28/4-1BB) to increase T-cell proliferation, cytotoxicity, and survival rates (13). Inducible second-generation transgenic proteins, such as the cytokine interleukin-12 (IL-12), were added to the fourth generation of CAR-T cells, also called T cells redirected for universal cytokine-mediated killing (TRUCKs), to improve antitumor activity (14). The design of the fifth generation of CAR is ongoing. Compared with the previous generations, fifth-generation CARs have been proven to have the ability to reactivate the immune system and maintain durability (15). They include the backbone chain of second-generation CARs and an added IL-2 receptor domain between the CD3 and CD28 signaling regions. Fifth-generation CARs are designed to simultaneously activate TCR, the costimulatory domain CD28, and cytokines.

CAR-T-cell therapy has achieved great success in treating hematological malignant tumors. To date, six CAR-T-cell therapies have been approved by the US Food and Drug Administration (16), and five have been approved by the Chinese National Medical Products Administration (NMPA) (**Table 1**).

**Table 1:** CAR-T-cell therapies marketed in China and the United States.

Listing country	Product name	Corporation	Approval time	Indication	CR	Target
USA	Kymriah (Tisagenlecleucel)	Novartis Pharma AG	2017.8.30	Precellular acute lymphocytic leukemia: recurrent or refractory diffuse large B-cell lymphoma	Complete response (CR):>90%	CD19
USA	Yescarta (Axicabtageneciloleucel)	Kite Pharma	2017.10.18	Recurrent or refractory diffuse large B-cell lymphoma: Recurrent or refractory follicular cell lymphoma	non-Hodgkin lymphoma Complete response (CR):51%	CD19
USA	Tecartus (Brexucabtagene autoleucel)	Kite Pharma	2020.7.24	Recurrent or refractory mantle cell lymphoma	mantle cell lymphoma Complete response:67%	CD19
USA	Breyanzi (Lisocabtagene maraleucel)	Juno Therapeutics	2021.2.5	Recurrent or refractory diffuse large B-cell lymphoma	Complete response (CR):54%	CD19
USA	Abecma (Idecabtagene vicleucel)	Bristol-Myers Squibb and bluebird bio, Inc	2021.3.26	Recurrent or refractory multiple myeloma	Complete response (CR):28%	BCMA
USA	Carvykti (ciltacabtagene autoleucel)	Legend Biotech Corporation	2022.2.28	Recurrent or refractory multiple myeloma	Complete response (CR):78%	BCMA
China	Axicabtagene Ciloleucel	Fosun Kite Biotechnology Co., Ltd	2021.6.22	Recurrent or refractory diffuse large B-cell lymphoma		CD19
China	Relmacabtagene autoleucel	JW Therapeutics (Shanghai) Co., Ltd.	2021.9.7	Recurrent or refractory diffuse large B-cell lymphoma		CD19
China	Equecabtagene Autoleucel	Nanjing IASO Biotherapeutics Co., Ltd.	2023.6.30	Recurrent or refractory multiple myeloma		BCMA
China	Inaticabtagene Autoleucel	Juventas Cell Therapy Ltd.	2023.11.7	Recurrent type B acute lymphoblastic leukemia		CD19
China	Zevorcabtagene Autoleucel	CARsgen Therapeutics Co. Ltd.	2024.2.23	Recurrent or refractory multiple myeloma		BCMA

CD19-targeted CAR-T cells are the first type of cell therapy with genetic engineering components approved in the US (17). CAR-T-cell therapy also has unique advantages in treating noncancerous diseases because of its excellent clinical effects (18–20). Experimental results suggested that the next major developments could be in areas beyond cancer, including autoimmune diseases, infectious diseases, and senescence-associated diseases, in which CAR-T cells will become widely applied. However, many problems with CAR-T-cell therapy exist in clinical practice (3, 8, 21). In many clinical trials, T lymphocytes are activated and amplified rapidly after CAR-T-cell infusion, causing excessive cytokine cascade release and finally leading to cytokine release syndrome (CRS) (22). Moreover, resistance triggered by loss of target is a key issue in CAR-T-cell therapy (23, 24).

There is still no CAR-T-cell therapy approved for solid tumor treatment in clinical practice because of the challenging characteristics of these tumors, such as loss of target antigen, an inhibitory tumor microenvironment (TME), and failed CAR-T-cell therapy caused by poor infiltration, a lack of tumor-killing ability and low durability (16, 25–28). In addition, the time-consuming and expensive nature of the treatment also presents challenges. A variety of strategies and methods have been used to overcome these hurdles, including arming CAR-T cells by knocking out PD-1 expression or secreting cytokines/chemokines and combining CAR-T-cell therapy with other therapeutic methods (29, 30). To date, over 700 clinical trials for CAR-T-cell therapy are underway (31), many of which focus on solid tumors.

Previous reviews have comprehensively discussed the problems faced by CAR-T-cell therapy, such as those in solid tumor treatment, resistance, and toxicity (2, 8, 24, 32). On this basis, here, we first summarize the mechanism of CAR-T-cell therapy in cancer treatment. Next, we analyze the challenges that CAR-T-cell therapy is facing, progress, and existing complications in the treatment of solid tumors and noncancerous diseases. Finally, we look ahead to the future development of CAR-T-cell therapy, proposing some viable and promising solutions to address various challenges. We also provide conclusions and recommendations, hoping for valuable insights for CAR-T-cell therapy.

## 2 Killing mechanisms of CAR-T-cell therapy

Normal T cells bind to target cells before killing them, forming immune synapses in the binding region. Similarly, CAR-T cells target tumor cells and form similar immune synapses, but their structure is slightly different from that of T cells, making the signaling pathway and triggering time slightly different (9). After CAR-T cells bind to tumor cells through immune synapses, they kill tumor cells through three mechanisms.

Initially, CAR-T cells secrete perforin and granzymes. Perforin can “dig holes” on the tumor cell surface, after which granzymes are transported into the tumor cells, damaging them directly or inducing cell apoptosis, which is considered the major killing mechanism of CAR-T cells (33). To ensure the precise killing of target cells, granzymes are anchored to the microtubes. After the formation of immune synapses, granzymes migrate to the interface, fuse into the plasmalemma of the center supramolecular activation cluster (cSMAC) (34), and are then released into the synaptic cleft by vesicles. In the synaptic cleft, perforin induces the formation of pores on the target cell membrane to promote the entry of proapoptotic granzymes. Upon entering the cytoplasm of target cells, granzymes can induce cell apoptosis by cleaving key substrates. The effects of perforin and granzymes are dependent on Ca^+^ (35).

CAR-T cells can also induce apoptosis through the Fas/FasL (CD95L) pathway (23). FasL is a cytokine that can bind to the death receptor TNFRSF6/FAS. It induces apoptosis triggered by cytotoxicity during T-cell development. The Fas/FasL pathway participates in immune cell homeostasis in nonpathogenic cases. The Fas/FasL pathway is calcium independent. Tschumi et al. reported that CAR-T cells can utilize this pathway to mediate tumor killing (36, 37). After CTL cells recognize target cells, the FasL expressed at high levels on the cell surface recognizes Fas on the target cell surface, triggering the apoptosis program inside the target cell through Fas and leading to programmed cell death of the target.

FasL is a homotrimer. Once it binds to the trimeric ligand, Fas recruits the Fas-associated protein with a novel death domain (FADD) in the cytoplasm through the death domain of its intracellular segment. The amino terminus of FADD contains a death effector domain (DED), which interacts with the DED domain of Caspase-8 to recruit Caspase-8 to the Fas region, forming a death-induced signaling complex (DISC) (38). The Caspase-8 proenzyme is activated and self-cleaves to form active Caspase-8. Activated Caspase-8 can activate downstream Caspase-3 to form mature Caspase, which then mediates cell death by cleaving over 500 cell substrates, effectively executing the apoptotic program (39). CD30 is a membrane-protein receptor on activated lymphocytes and is a member of the tumor necrosis factor receptor superfamily (40). CD30 CAR-T cells not only target CD30^+^ embryonal carcinoma (EC) cells through CAR-T cells but also eliminate surrounding CD30-ECs through Fas/FasL interactions. The Fas/FasL interaction between tumor cells and CAR-T cells can be used to reduce tumor escape caused by heterogeneous antigen expression or to increase the antitumor activity of CAR-T cells.

In addition to these two mechanisms, CAR-T cells can also secrete specific cytokines that promote CAR-T-cell competence, alter the TME, and further enhance antitumor activity. In a prostate cancer model, blocking TGF-β in T cells increased the ability of the cells to infiltrate, proliferate, and mediate antitumor responses. The efficacy of CAR-T cells can be increased through dominant negative TGF-β RII (dnTGF-β RII). CAR-T cells can specifically eliminate advanced tumors expressing prostate-specific membrane antigen (PSMA) (41). Boulch et al. reported that the main mechanism by which anti-CD19 CD4^+^ CAR-T cells eliminate tumors is the production of interferon (IFN)-γ rather than perforin, which mediates cytotoxicity. CAR4 T (CD4^+^ CAR-T) cells form high concentrations of IFN-γ in the tumor microenvironment to eliminate IFN-γ-sensitive tumor cells at the distal end. The intrinsic sensitivity of tumor cells to the proapoptotic effects of IFN-γ is the main determinant of CAR4-T-cell therapy (42).

Abnormalities in certain signaling pathways make solid tumors more resistant to CAR-T cells than hematologic cancers are. Larson et al. reported that glioblastoma and other solid tumors are more resistant to CAR-T cells owing to the loss of genes in the IFN-γ receptor (IFNγR) signaling pathway (IFNGR1, JAK1, or JAK2). However, the absence of this pathway does not make lymphoma cell lines insensitive to CAR-T cells (43). Tregs in the TME downregulate the type I interferon (IFN1) receptor IFNAR1 on CD8^+^ CTL through ADP−ribose polymerase family member 11 (PARP11). PARP11 is induced and overactivated in response to adenosine, thereby promoting the accelerated ubiquitination and degradation of IFNAR1. Therefore, partial INFAR1 deficiency disrupts CTL cytotoxicity and reduces their survival ability (44).

## 3 CAR-T cells beyond cancer

**Figure 3:**
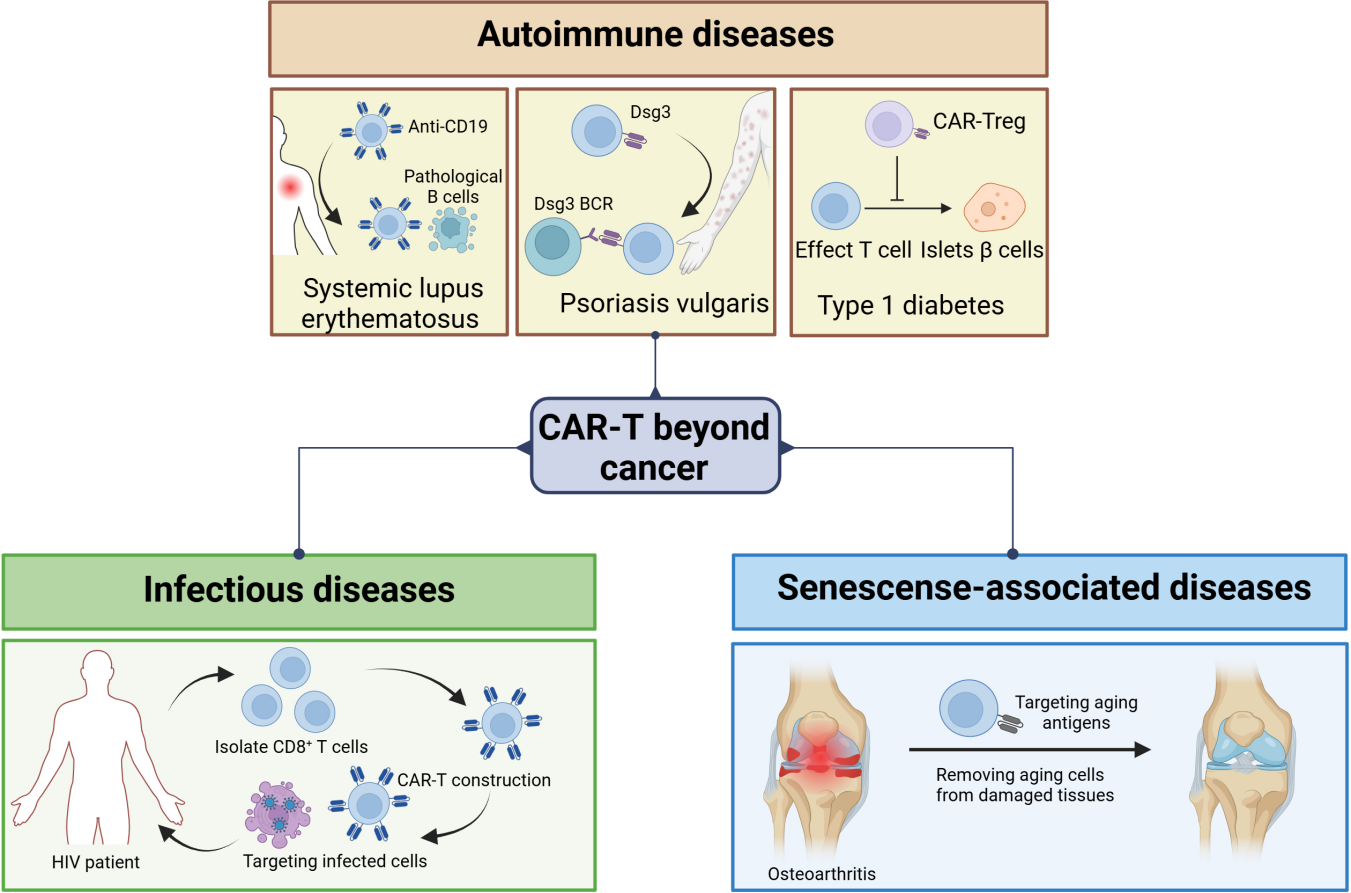
CAR-T cells for the treatment of noncancerous diseases. CAR-T cells are emerging in the field of noncancer disease treatment. CAR-T-cell therapy is not limited to cancer treatment. It has demonstrated significant potential in multiple areas, including autoimmune diseases, chronic infectious diseases, and diseases associated with aging. In autoimmune diseases, targeting CD19 can be used to treat SLE, and targeting Dsg3 can be used to treat PV, while CAR-Tregs can be used to treat type 1 diabetes. In infectious diseases such as AIDS, CAR-T-cell therapy is a potential way to clear host cells. With respect to senescence-associated diseases, CAR-T cells promote longevity by removing aging cells.

The success of CAR-T-cell therapy in the treatment of hematological cancers has sparked attempts to expand CAR-T-cell therapy to other therapeutic fields. To achieve significant therapeutic effects in cancer, almost all tumor cells must be eliminated. Moreover, in most other diseases, only a portion of pathological cells need to be cleared to achieve the therapeutic goal. Similarly, the TME is an important obstacle to CAR-T-cell therapy for cancer, but in most other diseases, target cells do not exist in environments similar to the TME and are more accessible (18, 45). CAR-T cells have been applied to autoimmune diseases (46–48), infectious diseases (49–51), senescence-associated diseases (52), and other fields (**Figure 3**, **Table 2**).

**Table 2:** CAR-T therapy for other diseases.

Type	Disease	CAR cell	Target	Report time
Autoimmune disease	Systemic lupus erythematosus	CAR T	CD19	2022.10
Autoimmune disease	Psoriasis vulgaris	CAAR T	Dsg-3	2016.6
Autoimmune disease	Type 1 diabetes	CAR Treg	InsB-I-Ag7	2019.9
Infectious diseases	AIDS	CAR T	HIV Host cell	2024.5
Senescence-associated disease	Senescence	CAR T	uPAR	2020.6

### 3.1 Autoimmune diseases

Systemic lupus erythematosus (SLE) is an autoimmune disease in which the body recognizes its own antigens as foreign antigens, leading to the activation of its own effector B and T cells. This type of attack on normal cells may lead to fatigue, inflammation, and, in severe cases, even death. In clinical trials, some therapeutic effects have been achieved by targeting B cells, but their efficacy is very limited because severe SLE is not easy to treat and still requires the use of drugs for maintenance after treatment (45). CAR-T cells targeting CD19 have shown the ability to eliminate pathological B cells in cancer; thus, researchers propose that CAR-T-cell therapy may have applications in the treatment of SLE. Mackensen et al. reported the use of CAR-T-cell therapy to treat 5 patients diagnosed with SLE (53). CD19 CAR-T cells were used to target B cells in SLE patients, and in all five patients, CAR-T-cell infusion improved their condition after the discontinuation of immunosuppressive drugs.

Psoriasis vulgaris (PV) is a life-threatening autoimmune disease caused by autoantibodies against Desmoglein3 (Dsg3). In PV, pathogenic memory B cells express anti-Dsg3 B-cell receptors (BCRs). Thus, targeting the elimination of anti-Dsg3 memory B cells should be able to cure PV without the risk of universal immune suppression. Ellebrecht et al. created a chimeric autoantibody receptor (CAAR) (46), using their own Dsg3 as the extracellular domain of CAAR to enable T cells to kill autoimmune B cells in the PV. CAAR T cells are a targeted therapy for antibody-mediated autoimmune diseases, which may generate long-term memory CAAR-T cells and potentially cure the disease.

Type 1 diabetes is an autoimmune disease characterized by the destruction of islet β cells by autoeffector T cells, leading to reduced insulin secretion and dysregulation of blood sugar. Inhibiting this autoimmune response is the main goal of the prevention and treatment of type 1 diabetes. Therefore, targeting regulatory T cells (Tregs) for therapy is a major area of focus (48). Spanier et al. developed a “TCR-like” CAR derived from an antibody that recognizes the insulin B peptide (InsB) present in I-Ag7 (54, 55). After *in vivo* administration, InsB-I-Ag7 CAR Tregs markedly reduced the development of spontaneous diabetes in NOD mice. Designing more effective CARs that target appropriate antigens can enhance the functional and targeting properties of Tregs.

### 3.2 Infectious diseases

CAR-T-cell therapy can also be used to treat infectious diseases. AIDS is an epidemic caused by human immunodeficiency virus (HIV) infection and has caused millions of deaths in recent decades. Although combined antiretroviral therapy (cART) has made considerable progress in inhibiting HIV replication, it has not been able to eliminate cells that are latently infected with HIV, and infected individuals remain HIV-positive for life (51). Lifetime antiretroviral therapy is needed to maintain control over virus replication. Therefore, there is an urgent need for new treatment strategies to eliminate the virus in the host to treat AIDS.

Researchers generate and amplify CAR-T cells that target HIV-infected cells from the patient’s blood and then reinject CAR-T cells into the patient’s body for therapeutic purposes. CD8^+^ T cells were collected from HIV patients, and CAR gene transduction was performed. After verifying the specificity and effectiveness of anti-HIV therapy *in vitro*, HIV-specific CAR-T cells were reinjected into the patient’s body to kill cells infected with HIV. The cytotoxic T cell (CTL) response is a key component of host immunity against HIV infection (56). In addition to restraining HIV replication during acute infection, enhancing the HIV-specific CTL response before the virus is activated can lead to rapid and effective killing of infected cells (57). Owing to this strong selective pressure, HIV quickly acquires mutations to evade CTL recognition. Unless cART is begun in the early stages of HIV infection, the vast majority of latent viruses carry CTL escape mutations (58). Equipping CD8^+^ T cells with CARs that can recognize various HIV antigens is crucial for the treatment of HIV. After binding to HIV envelope proteins, these CARs can trigger T-cell activation, proliferation, and cytokine production *in vitro*. CD4 receptors are used to construct anti-HIV CAR-T cells (59). CD4 interacts with gp120 during HIV infection and has a natural high affinity for HIV. CAR-T cells, which are based on CD4 receptors, have been shown to have the same level of cytotoxicity as natural CTLs. However, despite the marked advantages of CD4 receptors, CAR-T cells based on CD4 receptors are susceptible to HIV infection, and further research is needed to identify safer and more effective design targets.

### 3.3 Senescence-associated disease

The abnormal accumulation of aging cells produces an inflammatory environment, leading to chronic tissue damage and resulting in various senescence-associated diseases, such as atherosclerosis and osteoarthritis. Therefore, removing aging cells from damaged tissues can alleviate the symptoms of these diseases and even promote longevity (60). CAR-T cells targeting aging cells have the ability to act as senolytics. The characteristic molecules expressed on the aging cell membrane serve as unique antigen markers that can be selected for CTL-mediated senolytic activity and clearance. Amor et al. conducted a study on CAR-T-cell therapy targeting urokinase-type plasminogen activator receptor (uPAR) in mouse models (60). uPAR is a cell membrane protein that is widely expressed during the aging process and is associated with extracellular matrix remodeling. During replication, oncogene induction, and toxicity-induced aging, uPAR is upregulated on the surface of aging cells (61). uPAR-specific CAR-T cells can effectively eliminate aging cells *in vitro* and *in vivo*. CAR-T-cell therapy for senescence-associated diseases shows great promise. However, many key issues still need to be addressed. Aging cells exhibit hysteresis, as their phenotype depends on various stress factors to which they are subjected before symptoms appear. Moreover, the aging phenotype is strongly heterogeneous owing to factors such as the tissue from which it originates and the functions it performs, and every step from cell experiments to mouse experiments and even to clinical applications faces more complex transformations (52). Therefore, it is necessary to continuously optimize and test CAR-T cells to clarify the mechanisms.

## 4 Challenges facing CAR-T-cell therapy

**Figure 4:**
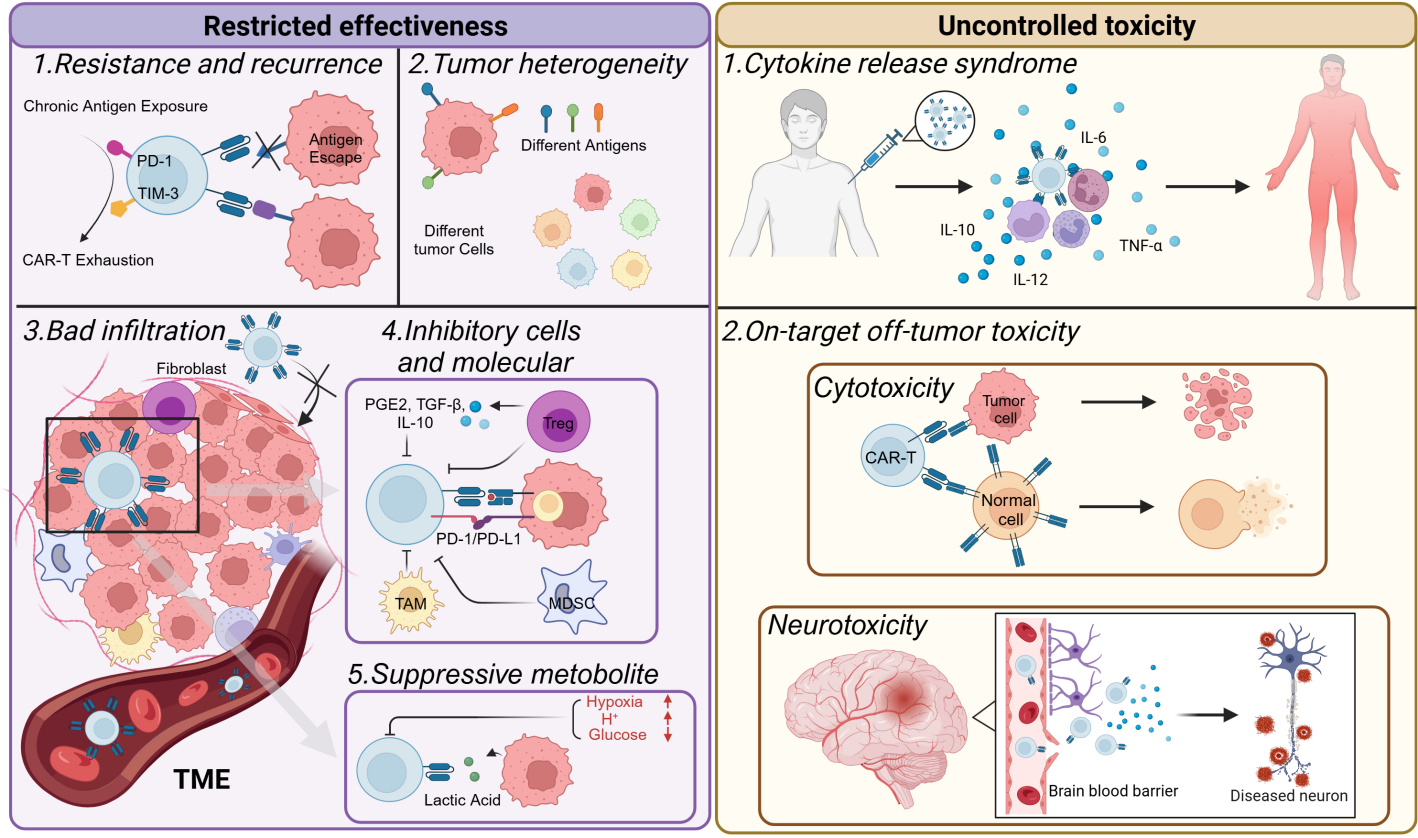
Challenges faced by CAR-T-cell therapy. CAR-T-cell therapy is constrained primarily by issues related to effectiveness and toxicity. CAR-T cells may become exhausted, resulting in short-lived resistance. This can lead to antigen escape in tumors, causing tumor recurrence. The heterogeneity of tumors also limits the efficacy of CAR-T-cell therapy. In the tumor microenvironment, various environmental changes occur. For example, it is often challenging for T cells to infiltrate solid tumors. In addition, immunosuppressive cells are recruited, inhibitory molecules are expressed, and metabolic reprogramming takes place. These factors collectively make it difficult to achieve a favorable prognosis when treating solid tumors. Moreover, CAR-T-cell therapy can sometimes produce excessive toxicity to the human body, inducing CRS or other on-target off-tumor toxicities and damaging normal cells in the body.

The use of CAR-T-cell therapy for hematologic counters faces many challenges. These include side effects induced by CAR-T cells, as well as problems related to resistance and recurrence. Although the successful application of CAR-T-cell therapy in treating hematologic cancers has spurred research into its potential use in solid tumors (2, 6, 11, 25, 62), it faces even more daunting challenges (**Figure 4**).

The tumor microenvironment (TME), characterized by its distinctive immunosuppressive features, is not only an accomplice in the development of resistance but also the primary factor contributing to the challenges faced by CAR-T-cell therapy in treating solid tumors. The TME is a complex entity that encompasses immune cells, stromal cells, blood vessels, cytokines, and the extracellular matrix. These various components interact with one another, continuously fueling tumor progression (63). This complex interaction sets the stage for the multiple roles that the TME plays in different stages of tumor development. In the early stages of tumor development, the TME promotes cancer cell growth, supports tumor colonization and invasion, and facilitates angiogenesis to overcome hypoxia and the acidic environment (64). The immunosuppressive TME can prevent CAR-T cells from effectively entering solid tumors. Moreover, it suppresses their immune effects, causing insufficient activation and exhaustion of CAR-T cells.

### 4.1 Resistance and recurrence

Although the efficacy of CAR-T-cell therapy is remarkable, resistance remains a crucial issue. Cancer recurrence after CAR-T-cell therapy is a major clinical challenge. In BLL patients treated with anti-CD19 or anti-CD22 CAR-T cells, the recurrence rate can reach 50% 12 months after infusion (24, 65). CD19 gene deletion and mutation may lead to the loss of the CD19 antigen on tumor cells, resulting in CD19-negative recurrence (66). CAR-T-cell resistance can be divided into primary resistance and secondary resistance. Primary resistance is due to a lack of response to CAR-T cells, whereas secondary resistance is characterized by cancer relapse after the initial brief response. The causes of resistance can be attributed to the dysregulation of CAR-T cells, the escape of tumor cells, and the inhibition of the TME (32).

From the perspective of immune cells, CAR-T-cell dysfunction is an important cause of resistance. Dysfunction of T cells may be caused by various factors, including long-term antigen stimulation, immune deficiency, or other undetermined factors (67, 68). Overactivation and high target affinity can exhaust T cells, reducing their cytotoxicity and persistence, potentially causing cell death. Exhausted T cells have low proliferation and cytotoxicity and high inhibitory receptor expression (PD-1^+^ TIM-3^+^). Since exhaustion is a tumor escape mechanism (69), reducing exhaustion and aging can promote CAR-T-cell function. Compared with endogenous CTLs, CAR-T cells have genetically modified antigen receptors that target tumor cells. However, their artificial design affects their effectiveness. CARs with CD28 costimulatory domains have poorer persistence than those with 4-1BB (24, 70). The manufacture of CAR-T cells requires a large number of healthy T cells from patients. Low-quality T cells can reduce the treatment efficiency of CAR-T-cell therapy. Chemotherapy can reduce patient lymphocytes and degrade product quality. Additionally, owing to age-related immune decline, young donor-derived CAR-T cells perform better than do those from elderly donors. Patients with advanced cancer may also be unable to provide high-quality T cells to manufacture CAR-T cells (71).

Tumor cells have multiple ways to escape immunity and develop resistance, and one of the most common mechanisms is the loss or downregulation of target antigens on tumor cells. Antigen escape, mutation, downregulation, or loss can lead to tumor cells evading recognition and clearance by the immune system, reducing the therapeutic effect of CAR-T-cell therapy and causing tumor recurrence (24, 72). CD19 CAR-T-cell primary resistance occurs in 10–20% of ALL patients; in a Philadelphia Children’s Hospital trial, 20 of 55 ALL patients experienced recurrence, 13 (24%) of whom were CD19 negative (73). Antigen loss or escape may become a major hurdle to cancer treatment, as there is greater heterogeneity in target antigen expression (66). Tumor cells can have genetic mutations that change their antigen structure, so they are sometimes unrecognizable by CAR-T cells. They can also evade by downregulating or losing antigens. There is also non-antigen-deficient resistance. In 2020, Singh et al. proposed that death receptor signaling dysfunction in cancer cells directly reduces CAR-T-cell killing (74). In ALL cells that resist CAR-T-cell attack, genes associated with activating cell death pathways (FADD, BID, CASP8, and TNFRSF10B) are missing, whereas genes required to resist cell death pathways (CFLAR, TRAF2, and BIRC2) are enriched. In the presence of antigens, prolonged survival of cancer cells can cause T-cell dysfunction. This effect is amplified when CAR-T-cell dysfunction is induced. This mechanism seems to rely on two stages: initial resistance to death receptor-driven killing, followed by antigen-driven CAR-T-cell dysfunction. The dysregulation of death receptor signaling in ALL leads to CAR-T-cell failure, further exacerbating the disease and promoting the development of resistance.

The tumor microenvironment plays a significant role in driving the development of resistance to CAR-T-cell therapy. Within the tumor microenvironment, inhibitory immune cells such as myeloid-derived suppressor cells (MDSCs), tumor-associated macrophages (TAMs), and regulatory T cells (Tregs) contribute to CAR-T-cell exhaustion (32). For example, Tregs usually express the inhibitory molecule PD-L1. PD=L1 can then directly inhibit the function of CAR-T cells, thereby reducing their ability to effectively target and eliminate tumor cells. Moreover, immunosuppressive cells and tumor cells secrete inhibitory cytokines, such as IL-10, IL-4, and TGF-β. These cytokines can reduce the effector function of CAR-T cells. This reduction in function further contributes to the development of resistance to CAR-T-cell therapy (75–77). It is crucial to further understand the mechanism of CAR-T-cell resistance and identify which patients are most likely to experience recurrence to optimize CAR-T-cell therapy.

### 4.2 Tumor heterogeneity

Tumor cells from different sources exhibit distinct morphological and phenotypic features, which is also one of the reasons solid tumors are difficult to treat. In cancer treatment, identifying ideal target antigens is crucial for the effectiveness of therapies. The first major difference between solid tumors and hematologic tumors is that it is more difficult to find ideal target antigens in solid tumors (16). The susceptibility of CAR-T cells to tumor-associated antigens (TAAs) determines the difficulty with which CAR-T cells can accurately recognize solid tumors. Moreover, owing to the loss of antigens and the absence of MHC presentation, solid tumors are difficult to target, resulting in extremely high heterogeneity of tumor cells. In addition, solid tumors exhibit TAA heterogeneity between tumor types (primary and metastatic) and patients with the same cancer (78). This high heterogeneity of solid tumors impacts immunotherapy efficacy, as the immune targets are limited to specific cells, hindering widespread killing.

Despite these challenges, researchers have explored potential target antigens to improve the treatment of solid tumors. EGFRvIII, a tumor-specific antigen (TSA) for CAR-T cells, is expressed only on human tumor cells and not on normal cells (79). CAR-T cells targeting EGFRvIII can accurately target tumor cells, which can improve therapeutic efficacy and reduce toxicity. However, the situation of target antigens in solid tumors is more complex. The shortage of TSA severely limits the use of CAR-T-cell therapy to treat solid tumors, as solid tumors rarely express TSA, which is different from hematological cancer, which commonly expresses TSA-CD19 (80). Therefore, targeting TAAs is an alternative method to overcome the shortage of TSAs. For most solid tumors, the most common antigens are TAAs, which are expressed at high levels on the tumor surface but also at low levels in normal tissues (81). TAAs are used in most ongoing clinical trials of CAR-T-cell therapy for solid tumors. Notably, CAR-T cells targeting TAAs are likely to cause damage to normal tissues, and potential risks should be fully evaluated before their use. With the continuous development of new technologies, high-throughput sequencing and other methods have enabled us to obtain more information about patients, including their specific mutation sites. This makes it possible to screen for new antigens and apply CAR-T-cell therapy to target new antigens in the future, potentially revolutionizing the treatment landscape of solid tumors.

### 4.3 Difficulties in CAR-T-cell infiltration

Even if solid tumor surface target antigens are present, CAR-T-cell trafficking into tumors is needed. In hematologic tumors, circulating CAR-T cells can directly damage tumor cells. However, in solid tumors, multiple obstacles must be overcome to enable infiltration (82). It is difficult for CAR-T cells to penetrate tumor tissue through the blood system of solid tumors. Solid tumors have unique histopathological features, such as concentrated blood vessels (83), tumor-associated fibroblasts, and myeloid cells that form the extracellular matrix (ECM). These features, in turn, contribute to the difficulty of T-cell infiltration into solid tumors. Although these features are beneficial for the growth of solid tumors, they increase the difficulty of T-cell infiltration at the tumor site, preventing continuous contact between T cells and tumor cells, which is necessary for T cells to exert their cytotoxic and antitumor effects. Ideally, the entry of T cells into the TME is a gradual process: tumor cells die and release antigens. Antigen-presenting cells (APCs) process and present tumor antigens. Interactions between APCs and T cells lead to T-cell activation, after which T cells reach the tumor site through the blood system, killing tumor cells by recognizing tumor antigens and releasing more antigens, resulting in a cascade amplification effect (84).

The most direct cause of T-cell infiltration disorders is insufficient T-cell recognition, which is often due to a lack of tumor antigens (85). After identifying tumor antigens, the antigens are processed, and the corresponding antigen peptide MHC-I class complexes on their surfaces are expressed. However, the absence of tumor antigens results in CAR-T cells being unable to effectively recognize tumor cells. In addition, changes in APC surface molecules, such as downregulation of MHC-I, limit the presentation of antigen peptide MHC-I complexes (84). Lysosomes are also associated with reduced infiltration of CD8^+^ T lymphocytes. In pancreatic ductal adenocarcinoma (PDAC), the autophagy-related receptor NBR1 induces the degradation of MHC-I on the surface of tumor cells, which further affects the T-cell response (86). These findings indicate that defects in the tumor antigen processing and presentation pathways inhibit T-cell initiation and hinder the effectiveness of cancer immunotherapy.

Tumor blood vessels are crucial for T-cell infiltration, but in many solid tumors, these vessels are twisted and diseased, especially in the tumor core (87). Damage to tight junctions and increased permeability lead to hypoxia, acidosis, and necrosis, inhibiting T-cell function and antitumor immunity. In the TME, cells secrete tumor angiogenic factors. Vascular endothelial growth factor (VEGF) regulates tumor angiogenesis by binding to VEGFR1-3. Anti-angiogenic therapy targeting VEGFR can normalize the tumor vasculature and be combined with CAR-T-cell therapy to increase infiltration (88). As a hallmark of cancer, hypoxia is caused by increased oxygen demand triggered by tumor cell proliferation and insufficient blood supply by angiogenesis (89–91). Hypoxia inducible Factor 1 (HIF1) is a hypoxia-activated transcription factor. Hypoxia inhibits T-cell infiltration by recruiting immunosuppressive cells. CCL28 and VEGF, which are induced by CCL28 and VEGF, affect angiogenesis and T-cell trafficking. Moreover, under hypoxic conditions, in combination with TGF-β, the expression levels of CD39 and CD73 in tumor tissue are upregulated. CD39 and CD73 convert ATP to adenosine, which binds to A2AR, inhibiting the production of cytokines such as IL-2 and T-cell development and proliferation (92). Hypoxia and TGF-β can ultimately upregulate the expression of CD39 and CD73 in tumor tissue. The sequential conversion of ATP to extracellular adenosine is catalyzed by CD39 and CD73. Adenosine binds to the adenosine A2A receptor (A2AR) and inhibits the production of cytokines such as IL-2, restraining the development and proliferation of T cells (93, 94).

In addition, the lack of chemokine expression involved in T-cell infiltration in tumor tissue and the presence of dense ECM in solid tumors lead to a reduced ability of CAR-T cells to migrate and invade tumor cells (95). The transport of CTL cells to the tumor site can be affected by the interaction between some chemokine receptors on CTL cells and their corresponding chemokines. The lack of some chemokines, including CXCL9, CXCL10, CCL4, and CCL5, has been reported to lead to the inhibition of T-cell infiltration (84). However, some chemokines, such as CXCL12, are unfavorable for T-cell infiltration into tumors. Stromal cells, especially cancer-associated fibroblasts (CAFs), are the main producers of CXCL12. CXCL12 produced by CAFs can mislead CTLs into the extracellular matrix of the tumor and prevent them from entering the tumor (96). Moreover, high expression of CXCL8 is associated with a decrease in the number of T cells in tumors, an increase in neutrophil and monocyte infiltration, and a limited response to immune checkpoint inhibitors (ICIs) ( (97). These results reveal the regulatory role of chemokine receptor and ligand interactions in CTL homing to the TME.

### 4.4 Inhibitory immune cells and molecules

Solid tumors typically contain numerous immunosuppressive cells, such as regulatory T cells (Tregs), myeloid suppressive cells (MDSCs), and tumor-associated macrophages (TAMs). Tregs are a special subgroup of CD4^+^ T cells that are responsible for suppressing immunity to prevent certain types of physiological damage caused by Th cell overactivation (98). In malignant tumors, Tregs may inhibit immune cell-mediated antitumor responses. In several cancers, including breast cancer, melanoma, and lung cancer, the increase in tumor-invasive Tregs is related to the low survival rate of patients (99). MDSCs are derived from myeloid progenitor cells, including immature macrophages, immature granulocytes, and immature dendritic cells (DCs). MDSCs have been shown to aggregate in tumors as T-cell inhibitors, secrete various inhibitory cytokines, and upregulate the expression of certain immunosuppressive molecules (100). In addition to being phagocytic and antigen-presenting cells, macrophages secrete various factors with immune-supportive or immunosuppressive functions. In the TME, these cells are called TAMs, and tumor malignancy is positively correlated with TAM enrichment in different solid tumors (101). These immunosuppressive cells help to protect solid tumor cells from being killed by the host immune system.

Tumor-derived factors are a class of soluble factors that inhibit the efficacy of CAR-T-cell therapy for solid tumors. They are usually secreted by immunosuppressive cells, such as Tregs, which can secrete large amounts of PGE2, TGF-β, IL-10, and other substances that inhibit T-cell proliferation. TGF-β plays an important role in restraining antitumor responses. TGF-β can downregulate CD8^+^ effector T-cell function and promote Treg maturation (83). Therefore, the function of CD8^+^ T cells can be improved by binding to TGF-β, which means that in CAR-T-cell therapy, the population, infiltration, and persistence of T cells can be increased to boost the antitumor response.

The inhibitory function of immune responses is induced by immune checkpoint ligands, which are usually overexpressed in solid tumors. These ligands can induce the expression of immunosuppressive molecules or their receptors, including PD-L1/PD-1, T lymphocyte immunoglobulin mucin 3 (TIM-3), indoleamine 2,3-dioxygenase 1 (IDO-1), lymphocyte activation gene 3 (LAG-3), and cytotoxic T lymphocyte-associated protein 4 (CTLA-4) (62). Their inhibitory effects on T lymphocyte activation lead to tumor immune escape. For example, PD-L1 binds to PD-1, suppressing CAR-T-cell activation and increasing tumor immune tolerance. TIM-3 contributes to T-cell exhaustion in the TME. Recent immunotherapy research has focused on blocking these checkpoints to alleviate immune suppression and restore immune function. Combining immune checkpoint inhibitors such as CTLA-4 and PD-1 with CAR-T cells targets immunosuppressive cells in the TME (82). PD-L2, which is expressed on the surface of DCs, macrophages, mast cells, and some B cells, is the second ligand, after PD-L1, that can bind to PD-1. PD-L2 is also significantly expressed in the TME of renal cell carcinoma (RCC) and lung squamous cell carcinoma (LUSC). The coexpression of PD-L1 and PD-L2 in tumor cells significantly inhibits antitumor immune responses (102). In the future, the application of anti-PD-L2 monoclonal antibodies may overcome the limitations of anti-PD-L1 monoclonal antibody drugs.

### 4.5 Metabolism in the TME

The highly active metabolic pathways unique to tumor cells can cause significant changes in the composition of nutrients and other small molecules within the tumor microenvironment, which have critical impacts on immune responses. The high metabolic activity of tumor cells and the chaotic vascular system within the TME may lead to nutrient exhaustion and hypoxia, and this situation establishes metabolic competition between tumor cells and infiltrating immune cells (103).

Glucose restriction in the TME substantially affects T-cell responses. Owing to their highly active metabolic function, the ability of tumor cells to take up glucose is greatly increased, resulting in a low-glucose environment in the TME. Research has shown that low-glucose conditions (0.1 mM) inhibit the production of phosphoenolpyruvate (PEP), an intermediate product of glycolysis in T cells, disrupting calcium-dependent NFAT signaling and inhibiting T-cell proliferation, activation, and cytokine production, leading to a decrease in T-cell survival ability (104).

Hypoxia is caused by an imbalance in the oxygen consumption of rapidly proliferating tumor cells, coupled with an insufficient oxygen supply caused by abnormal tumor angiogenesis (105). Owing to their high density, tumor cells exceed the limit of oxygen diffusion from capillaries (106). Therefore, diffusion limitations, leakage, and deformities of the tumor vascular system lead to a hypoxic environment. At the molecular level, the adaptation of tumor cells to the hypoxic TME is largely mediated by the hypoxia inducible factor (HIF) family (107). Although tumor cells can adapt to the hypoxic environment, immune cells are inhibited under these conditions. *In vitro* experiments have shown that, compared with normoxia, low concentrations of oxygen significantly reduce the proliferation and function of T lymphocytes and promote their apoptosis (108). Hypoxia can delay the differentiation of effector T cells and reduce the generation of effector T cells and cytokines such as IFN-γ and IL-2. Accordingly, in the hypoxic environment of the TME, CAR-T cells are notably restrained and have difficulty functioning normally.

Many metabolites produced by cancer cell metabolism can also affect infiltrating T cells. *In vitro* studies of CD8^+^ T cells in mice and humans have shown that elevated levels of extracellular lactate and H^+^ in the TME can inhibit T-cell proliferation, survival, cytotoxicity, and cytokine production (103). Tumor cells tend to choose the metabolic mode of glycolysis, leading to the accumulation of lactate in the microenvironment and acidification of the TME. Changing cell markers and preventing cell differentiation can lead to carcinogenesis, increasing the survival and proliferation of tumor cells, stimulating angiogenesis, and inhibiting immune responses by altering several immune infiltrating cells. The immunoregulatory role of lactic acid has drawn much attention. It affects T-cell activity and CAR-T-cell efficacy by promoting the infiltration of immunosuppressive cells such as TAMs, MDSCs, and Tregs. Xiong et al. combined epigenetics with tumor lactate metabolism. Methyltransferase-like 3 (METTL3) regulates tumor metabolic reprogramming by controlling the expression of glucose transporters (GLUTs), lactate dehydrogenase (LDHA), and enolase 1 (ENO1) in tumor cells. High METTL3 expression in cancer (109) significantly increases glucose uptake and lactate production, enhances glycolysis, and promotes tumor growth (110). On this basis, lactic acid in the TME promotes the expression of METTL3 in TAMs through histone lysine lactylation (Kla) modification. On the other hand, lactic acid lactates the zinc finger domain of METTL3, promoting the methylation of JAK1 mRNA, which combines with YTHDF1 to improve translation efficiency and promote the activation of the JAK1-STAT signaling pathway, initiating the expression of downstream immunosuppressive molecules and resulting in immunosuppression (109). Immunosuppressive cells subsequently lead to poor therapeutic effects by inhibiting CAR-T cells.

### 4.6 Toxicity of CAR-T cells

Immunotherapy has unique toxicity that traditional cytotoxic chemotherapies or small-molecule inhibitor do not have (111–113). Patients receiving CAR-T-cell therapy can experience many potentially life-threatening toxicities, and CRS is one of the most clinically importance and dangerous. CRS occurs because of the high-level immune activation of immune cells, followed by the release of many inflammatory cytokines, which are most commonly observed in therapies involving T cells (8, 114, 115).

Researchers define CRS as “a supraphysiologic response following any immune therapy that results in the activation or engagement of endogenous or infused T cells and/or other immune effector cells. Symptoms can be progressive, must include fever at onset, and may include hypotension, capillary leak (hypoxia), and end-organ dysfunction (111).” The clinical manifestations of CRS involve multiple systems and are accompanied by life-threatening complications, including cardiac dysfunction, respiratory failure, kidney and liver failure, etc. Fever is usually the first sign of CRS (116). Low-level CRS manifests as an influenza-like illness characterized by fatigue, muscle soreness, etc. According to the severity of the disease, CRS is divided into 5 grades (111), ranging from grade 1 (fever at 38°C) to grade 5, which may result in death. After treatment with CD19 CAR-T-cell therapy, 54% to 91% of patients develop CRS, and 8.3% to 43% of patients develop severe CRS (22). In a clinical trial involving 75 patients receiving tisagenlecleucel (CD19 CAR-T) treatment, there was a brief increase in the serum IL-6, IFN-γ, and ferritin levels during the CRS period after infusion. These increases were often more pronounced in Grade 4 CRS patients than in lower-grade patients. Similar trends were also observed for other cytokines, including IL-10, IL-12, IL-1β, IL-2, IL-4, IL-8, and TNF-α. A brief increase in C-reactive protein was observed in most patients, but the variability was high (117).

IL-6 is an important cytokine that plays a crucial role in host defense by regulating immune and inflammatory responses. The IL-6/IL-6R signaling pathway is the main mediator of CRS occurrence; therefore, targeting IL-6 for anticytokine therapy is an effective treatment method to mitigate CRS symptoms. In classical intracellular signaling, IL-6 binds to IL-6R on the membrane and interacts with membrane glycoprotein 130 (gp130), which induces dimerization and leads to intracellular signal transduction, thereby activating and releasing inflammatory cytokines (118). Tocilizumab is a humanized monoclonal antibody against IL-6R that can competitively inhibit the binding of IL-6 to receptors and has been widely used to treat rheumatism. Tocilizumab has been reported to have little effect on the efficacy of CAR-T-cell therapy and relatively few substantial side effects. In a clinical study of patients receiving tisagenlecleucel treatment, tocilizumab was administered to 13 patients with cardiovascular dysfunction, with fever and tachycardia rapidly subsiding within 4 hours after treatment (112).

A significant challenge faced by CAR-T-cell therapy for solid tumor patients is that most candidate target antigens are typically coexpressed in healthy tissues, which poses a considerable risk of “on-target, off-tumor” toxicity (25). Multiple clinical trials have reported varying degrees of “on-target, off-tumor” toxicity (79, 119, 120). In these trials, CAR-T cells targeted both tumor cells and normal cells, leading to significant damage and severe side effects such as neurotoxicity.

Neurotoxicity is a common and potentially life-threatening adverse reaction to CAR-T-cell therapy. Neurological damage is often associated with CRS that occurs during or after CD19 CAR-T-cell therapy and, in rare cases, may threaten a patient’s life (121, 122).

The neurotoxicity caused by CAR-T cells is diverse, and patients may experience symptoms such as hallucinations, cognitive deficits, aphasia, speech disorders, seizures, and encephalomyelitis (123–128). Like CRS, neurotoxicity is classified into 5 grades according to severity, ranging from mild to severe. Neurotoxicity may occur simultaneously with certain symptoms of CRS (such as hypotension), but neurotoxicity may also occur in patients who do not exhibit typical CRS symptoms or after CRS subsides (129). According to reports, neurotoxicity typically first appears on the second day after CAR-T-cell infusion and can last until the third or fourth week (130), resulting in a highly variable course. Therefore, close monitoring of neurotoxicity is necessary during the use of CAR-T-cell therapy.

In a clinical trial, the manifestations of 25 adult patients with neurotoxicity syndrome who received CAR-T-cell therapy at Massachusetts General Hospital were described (131). This clinical trial involved 23 NHL patients and 1 ALL patient receiving CD19 CAR-T-cell therapy, along with 1 liver cancer patient receiving AFP-targeted CAR-T-cell therapy. Among the 25 patients, 12 (48%) experienced grade 1-2 neurotoxicity, and 13 (52%) experienced grade 3-4 neurotoxicity. Tocilizumab does not seem to reduce the severity of neurotoxicity. Instead, it may increase exacerbate it. Neurotoxicity is primarily treated with corticosteroids, and its symptoms are controlled (132). Although corticosteroids are widely used, the extent to which they affect the anticancer effects mediated by CAR-T cells is still unclear, and further clinical trials are needed to determine their usage standards.

The pathogenesis of neurotoxicity remains obscure (133). Severe neurotoxicity may be related to the levels of C-reactive protein, various serum cytokines, and other proteins, such as IL-2, IL-6, and IL-10, as well as TNF-α and Granzyme B (125, 133). When the blood−brain barrier is broken, CAR-T cells can enter the cerebrospinal fluid and may significantly increase cytokine levels in the cerebrospinal fluid (134), causing neurotoxicity. In 2020, Parker et al. proposed a possible neurotoxicity mechanism (135). CD19 has been reported to be expressed in brain cells that protect the blood−brain barrier. In mouse models, when CAR-T cells are infused into mice, even if the mice lack B cells, their brain blood−brain barrier permeability increases. In contrast, when human CD19 was used as a CAR-T-cell target, there was no significant change in blood−brain barrier permeability. Compared with other B-cell proteins, such as CD20, CD19 immunotherapy has a higher incidence rate of neurotoxicity. Therefore, they concluded that the expression of CD19 molecules on the surface of cells other than B cells (such as blood−brain barrier cells) is the fundamental cause of CAR-T-cell-induced neurotoxicity.

### 4.7 High cost of CAR-T cells

CAR-T-cell therapy, an emerging immunotherapy, has achieved significant therapeutic success, but its high cost makes it difficult for ordinary people to afford. In China, five CAR-T-cell therapies have been approved for the market, including Zevorcabtagene Autoleucel (BMCA), Axicabtagene Ciloleucel (CD19), Relmacabtagene Autoleucel (CD19), Equecabtagene Autoleucel (BCMA), and Inaticabtagene Autoleucel (CD19), priced at 1.15 million yuan, 1.2 million yuan, 1.29 million yuan, 1.166 million yuan, and 0.99 million yuan, respectively. How to solve the problem of high costs has always been a topic of concern for R&D personnel. The high price of CAR-T cells is caused by various reasons. First, CAR-T cells are personalized products, and each batch of CAR-T cells corresponds to only one patient; thus, it is not possible to reduce costs by treating more patients. Second, CAR-T-cell therapy involves complex production processes and treatment procedures with high-quality control, production, and management costs (136). The clinical trial cost of CAR-T-cell therapy is also very high, and in addition to treatment, there are additional considerations for health care costs (137), which all contribute to the high cost of CAR-T-cell therapy.

## 5 Future perspectives

**Figure 5:**
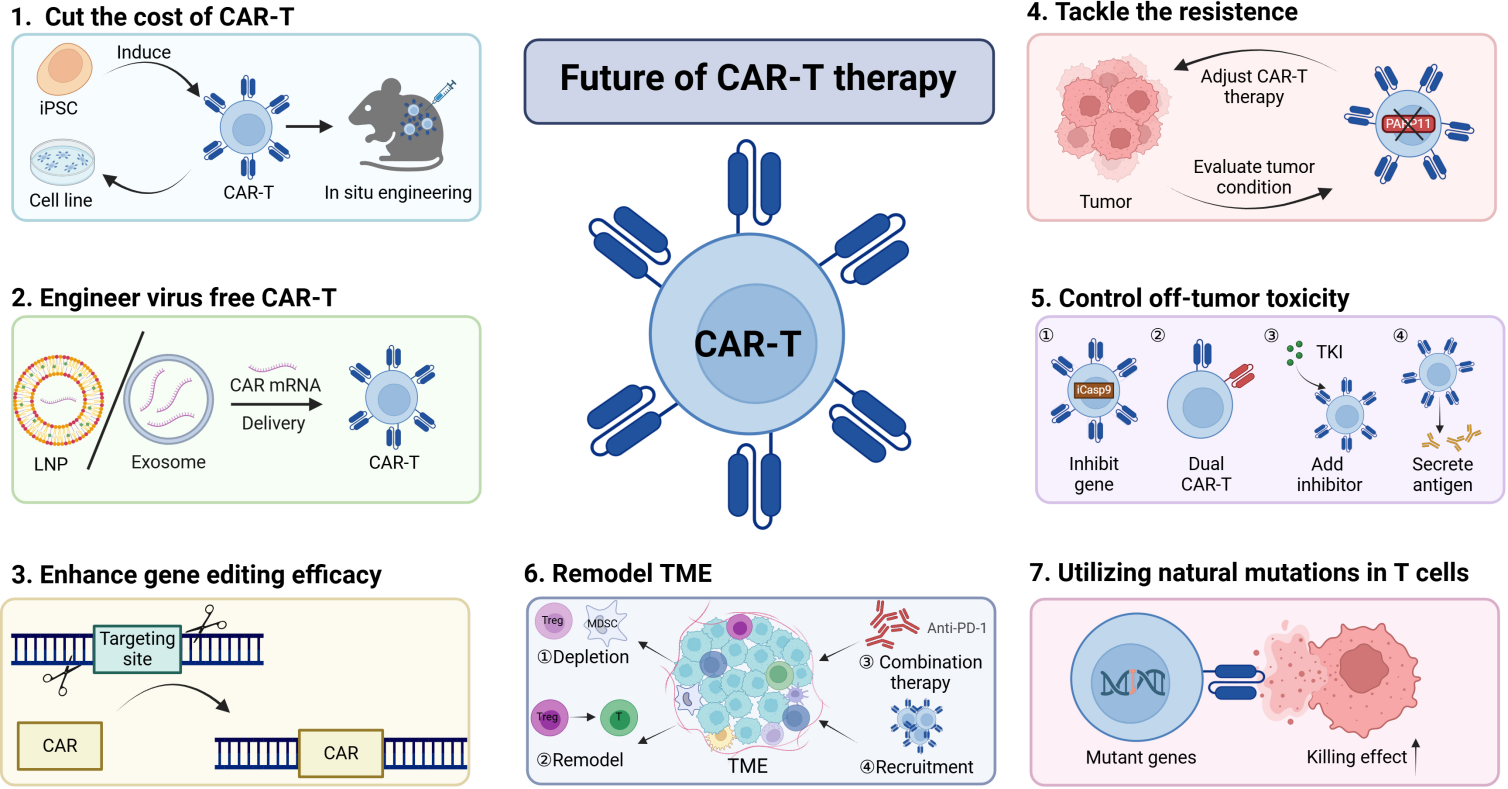
Harnessing multiple strategies to increase CAR-T-cell efficacy. The efficacy of CAR-T-cell therapy can be increased by employing various strategies.(1)The cost of CAR-T cells can be reduced by inducing CAR-T cells from iPSCs and cell lines or through *in situ* engineering approaches. (2)Virus-free engineering of CAR-T cells with LNPs or exosomes is an effective means to decrease genotoxicity.(3-4)The discovery of novel gene editing methods and the exploration of new targets have the potential to increase CAR-T-cell efficacy and overcome tumor resistance to CAR-T-cell therapy.(5)It is essential to control excessive toxicity by constructing new types of CAR-T cells.(6-7)Remodeling the TME or leveraging natural mutations can also contribute to the enhancement of CAR-T-cell efficacy. Collectively, these approaches can be utilized to improve CAR-T-cell therapies, offering promising avenues for the advancement of this field in the treatment of various malignancies.

Although CAR-T-cell therapy has achieved many exciting results, in future research, we still need to study the mechanisms more deeply and find solutions to the problems, including cost, specificity, resistance, toxicity, and TME inhibition (**Figure 5**).

### 5.1 Decreasing the cost of CAR-T-cell therapy

#### 5.1.1 Establishing cell lines

There are two main reasons CAR-T-cell therapy is expensive (1): The process is very cumbersome, involves multiple aspects, and requires a dedicated medical team (2). Currently, CAR-T-cell therapy is approved only for the treatment of certain tumors in the bloodstream, and its unsatisfactory effect on solid tumors greatly limits its large-scale application. Personalized therapy makes it difficult to reduce labor costs. At present, CAR-T-cells are produced mainly by autologous CAR-T-cell technology, in which T cells are collected from patients themselves and processed. If CAR-T cells are constructed into stable cell lines, it will become possible to prepare them in advance, ensure the timeliness of treatment, and achieve large-scale production, which will greatly reduce costs. By selecting appropriate host cells, constructing plasmid expression vectors containing CAR genes, and transfecting them into cells, we can screen stable expression cell populations using appropriate selection pressure and continuously culture them *in vitro*. Of course, there are some issues here, such as challenges in the process of building cell lines. Allogeneic CAR-T cells may lead to life-threatening GVHD. Moreover, these allogeneic CAR-T cells may be rapidly eliminated by the host immune system, reducing their antitumor activity and treatment outcomes.

#### 5.1.2 iPSCs

Human induced pluripotent stem cells (hiPSCs) can provide an unlimited source of T cells for the development of CAR-T cells, with the potential to produce ready-made T-cell products. The combination of iPSCs and CARs provides an encouraging opportunity for cancer treatment, simplifying cell therapy methods for cancer patients and greatly reducing costs. In this case, gene editing of T-iPSCs (iPSC-derived T cells) using CAR would be a promising strategy to obtain T-iPSCs without restriction and generate functional T cells with a defined phenotype for therapeutic purposes. In a previous study, Themeli et al. isolated peripheral blood mononuclear cells (PBMCs) from healthy donors and transduced them with two retroviral vectors (138). Under laboratory conditions, the transduced T cells produce T-iPSCs, which are then transduced using a lentiviral vector encoding CD19-28z-CAR. The transduced iPSCs differentiate into T cells expressing CAR and endogenous TCR. A CD19-T-iPSC line targeting the CD19 antigen was successfully generated in a mouse model of human CD19^+^ Burkitt lymphoma. *In vivo* cytotoxicity assays revealed strong antitumor activity and significant tumor regression.

However, the combination of T-iPSCs and CARs is still in its early stages of development and requires further experimental research (139). There are still challenges in the differentiation, maturation, and functionality of current iPSC-derived CAR-T cells, especially iPSC-derived T cells, which typically exhibit characteristics similar to those of gamma delta T cells and lack the powerful function of mature alpha beta T cells in the peripheral blood (140). iPSC-derived T cells are not considered readily available products. Owing to the presence of endogenous TCR or HLA mismatches, they cannot be used for third-party patients (138). The most common adverse reaction associated with TCR is GVHD, where donor T cells recognize host antigens as foreign antigens and subsequently destroy them.

### 5.2 *In situ* engineering

CAR-T cells produced *in vivo* provide an effective alternative immunotherapy for cancer treatment. This method requires a vector to deliver the CAR-encoding construct to T cells to express the CAR to eradicate the tumor. Therefore, an increasing number of studies have reported gene delivery systems for *in vivo* CAR-T-cell therapy based on viral vectors and polymer nanoparticles. Delivery methods based on biomaterials may change the manufacturing process of CAR-T cells, significantly reducing costs (141–143). Regulating the tumor microenvironment with biomaterials can also substantially increase the therapeutic effect of CAR-T cells against solid tumors. The *in situ* engineering of CAR-T cells can avoid the current need to extract autologous cells from patients, allows large-scale production, and facilitates storage and transport. Nanoparticles can be formulated and infused into patients when necessary. In terms of drug administration, *in situ* engineering can also save patients from the step of chemotherapy pretreatment (144). The modified CAR-T cells can also immediately recognize cancer cells *in vivo* and do not require additional ex vivo expansion steps. In one study, Smith et al. developed a polymer platform based on PBAE/PGA nanocarriers for the *in vivo* delivery of a CD19 CAR (145). The tumors were eradicated in approximately 70% of the mice treated, while the remaining mice showed notable regression, and an increase of 58 days in average survival time was observed. The therapeutic effect was comparable to that of CD19 CAR-T cells produced by infusion using standard methods *in vitro*. Fibrosis affects millions of heart disease patients. By delivering modified mRNAs into lipid nanoparticles (LNPs) that target T cells, transient antifibrotic CAR-T cells were generated *in vivo*, and CD5-targeted LNPs were injected into a heart failure mouse model to evaluate the efficacy of these reprogrammed CAR-T cells *in vivo*. The mRNA encoding CAR was effectively delivered to T lymphocytes, resulting in the *in vivo* generation of immediately effective CAR-T cells *in vivo*. Moreover, the accumulation of antifibrotic CAR-T cells in the spleen preserves target antigens, which can reduce fibrosis after injury and restore cardiac function (146).

### 5.3 Virus-free transduction

The majority of CAR-T cells are currently prepared through lentiviral transduction. Although lentiviruses can induce permanent expression of CARs, the uncertainty of the insertion site may lead to serious adverse reactions in patients receiving CAR-T-cell therapy (147). In addition to safety issues, there are various obstacles to the production and *in vivo* transfection of CAR-T cells by lentiviral transduction, including limited load, high cost, and *in vivo* immunogenicity. There is an urgent need for a safer and cheaper CAR-T-cell production strategy to replace lentiviral transduction.

mRNA transduction is a promising strategy for inducing transient CAR expression in T cells to avoid the adverse effects associated with viral vectors. Using mRNA to induce CAR expression has several advantages over viral transduction. First, mRNAs do not need to be integrated into the host cell genome, allowing CARs to be expressed in a short period of time, effectively avoiding the long-term risks related to CAR-T cells *in vivo*. Moreover, *in vitro* transcription is beneficial for optimizing the structure of CAR mRNA, making it easier for CAR proteins to be translated and expressed in T cells (148). mRNA-based CAR-T-cell therapy effectively kills tumor cells and achieves therapeutic results similar to those of lentivirus-mediated CAR-T-cell therapy. However, the most common requirement for mRNA delivery is electroporation, which may cause cytotoxicity (149). However, LNPs have been designed to deliver mRNA to T cells (150). This platform induces CAR expression at a level equivalent to electroporation with markedly reduced cytotoxicity. Furthermore, exosomes are phospholipid bilayer vesicles secreted by living cells into the extracellular microenvironment. Exosomes contain many bioactive molecules and, together with their corresponding receptors, can mediate intercellular communication (151). Owing to their biocompatibility, low immunogenicity, and ability to penetrate the blood−brain barrier, exosomes are potential RNA drug carriers. By utilizing engineered exosomes, CAR-T cells can be directly generated from peripheral blood mononuclear cells (PBMCs), providing a methodological reference for the development of safer and more cost-effective CAR-T-cell therapies in the future (152).

Virus-like particles (VLPs) are multiprotein structures that are very similar to viruses, but owing to the lack of a viral genome, they can be safely used. These VLPs originate from both enveloped and nonenveloped viruses, self-assemble from one or more viral structural proteins, and can spontaneously form particles close to the size and shape of the original virus. Genetic materials such as DNA or RNA can be packaged into VLP nanocages and delivered to target cells, demonstrating the potential of VLPs as gene editing vectors (153, 154). Engineered DNA-free virus-like particles (eVLPs) have been developed for packaging and delivering base editors or Cas9 ribonucleoproteins, which mediate efficient base editing in several mouse and human cells (155). For example, regarding v3 and v3b PE-eVLPs, a single injection of v3 PE-eVLPs can restore the expression of treatment-related proteins in the retina and partially salvage visual function in two genetic blindness mouse models (156). The use of VLPs instead of traditional viral vectors for gene editing delivery can increase the safety of CAR-T cells.

### 5.4 Improving the site specificity of gene editing

The emergence of CRISPR/Cas9 technology, which is simple, reliable, and effective, has increased the efficiency and specificity of gene editing. It has been used for sequence knock-in or knockout in mammalian genome editing. Cas9 nuclease, guided by s small guide RNA (sgRNA), induces double-stranded DNA breakage (DSB), which is repaired through nonhomologous end joining (NHEJ), leading to gene function loss. Compared with NHEJ, after double-stranded DNA is cleaved by sgRNA, homologous directed repair can deliver larger gene sequences to precise sites in the genome (157–159). The HDR process can perform precisely targeted nucleotide substitution at designated sites (160).

The current research focus is on the precise editing of CAR-T cells using the CRISPR/Cas9 system, which can install the required genes in the genome with or without the introduction of DSBs. These tools and strategies can be directly applied to negative regulatory factors of T-cell function, target genes to specific loci, and produce repeatable, safe, and effective universal CAR-T-cell products for cancer immunotherapy (158). The latest progress in CRISPR technology has enabled endogenous genes to target and intervene in human CAR-T cells, unleashing the therapeutic potential of CAR-T-cell therapy. Strategies based on CRISPR have been used to develop next-generation CAR-T cells (161). Guiding the CD19-specific CAR to the T-cell receptor alpha constant (TRAC) locus not only leads to uniform expression of the CAR in human peripheral blood T cells but also increases the efficacy of T cells. In a mouse model of acute lymphocytic leukemia, the edited cells were significantly superior to traditional CAR-T cells. These findings emphasize the potential of the CRISPR/Cas9 system in advancing immunotherapy (162). CRISPR/Cas9 technology can also be used to knock down genes related to T-cell exhaustion and restore the loss of epigenetic targets, with the potential to prevent or even reverse CAR-T-cell dysfunction. Targeting inhibitory receptors, transcription factors, or other mediators of CAR-T-cell dysfunction through gene editing can reactivate injected cell products (158).

Although HDR can be used to insert specific DNA templates for the precise restoration of DNA sequences, this pathway is characterized by limited efficiency and high rates of unexpected insertion or deletion mutations, which render the repair ineffective. In addition, relying on homologous recombination limits the range of targeted diseases (163). The CRISPR/Cas-mediated single base pair editing system has been designed to overcome these limitations. DNA base editors (BEs) include the catalytic fusion of damaged Cas nucleases and base-modifying enzymes, which act on single-stranded DNA (ssDNA) rather than double-stranded DNA (dsDNA). When the guide RNA binds to the target DNA site, the base pairing between the guide RNA and the target DNA strand causes a small segment of single-stranded DNA in the “R-loop” to shift. The DNA base in this single-stranded DNA is modified by deaminase. To increase the efficiency of gene editing in eukaryotic cells, the catalytic inactivation of nucleases creates a gap in the unedited DNA strand, inducing the use of the edited strand as a template to repair the unedited strand (164, 165). A study combined nonviral CRISPR/Cas9 nuclease-assisted knock-in and Cas9-derived base editing techniques to perform DSB-free knockout within a single insertion range, inserting CAR into the TRAC gene, as well as two knockouts to silence MHC I and MHC II expression. This method increases the site specificity of gene editing and can effectively generate edited CAR-T cells with a translocation frequency comparable to that of unedited T cells, thereby enhancing the safety of CAR-T cells (166).

Another type of gene editing tool, prime editors (PEs), is a new method that can expand the scope of precise DNA editing without donors. It can be used for all transition and transposition mutations, as well as small insertion and deletion mutations. PEs do not require double-stranded DNA breakage and can perform almost any substitution, small insertion, or small deletion in the DNA of living cells. Prime editing requires at least one programmable nickel enzyme and polymerase fusion, as well as an extended guide RNA that can specify target sites and template the required genome editing (167, 168). One study designed a powerful knock-in (KI) strategy using PEs, called primed microhomology-assisted integration (PAINT), which utilizes reverse transcription of single-stranded microhomology sequences to promote targeted KIs in different types of cells (169). An improved version, PAINT 3.0, maximizes editing efficiency and reduces the possibility of off-target editing. By using PAINT 3.0, a reporter gene can be transformed into a housekeeping gene, with an editing efficiency of up to 80%, which is more than 10 times greater than that of traditional homologous directed repair methods. PAINT 3.0 can efficiently target nonviral genomes in primary T cells and generate functional CAR-T cells with specific tumor-killing abilities. Overall, DNA base editing and prime editing tools can perform precise nucleotide substitutions in a programmable manner without the need for donor templates. Both DNA base editing and prime editing have significant potential as tools for the precise editing of CAR-T cells.

### 5.5 Counteracting resistance

Understanding the mechanisms of resistance can provide more appropriate treatment strategies to address this problem. B-cell maturation antigen (BCMA) is crucial for the normal function of plasma cells, so its absence is not common. Samur et al. determined through single-cell transcriptome analysis of bone marrow samples that the absence of BCMA is one of the mechanisms of resistance in CAR-T cells (170). The therapy achieved clinical effects during the initial infusion of CAR-T cells in the patient, but resistance developed later, manifested as recurrence and a lack of response to the second infusion of CAR-T cells. These findings emphasize that myeloma cells may still be able to survive without BCMA. Accordingly, CAR-T-cell therapy may result in the selection of BCMA-negative myeloma cells may be selected, leading to resistance. Therefore, it is possible to consider sequencing the BCMA locus before continuous BCMA-targeted therapy to fully evaluate the growth of rare BCMA-deficient myeloma cells and adjust treatment strategies in a timely manner.

The TME helps to evade antitumor immunity and generate resistance to treatment. Therefore, targeting certain key factors in the TME is a key approach for addressing CAR-T-cell resistance. Zhang et al. reported that TME factors such as regulatory T cells and adenosine downregulated the type I interferon (IFN1) receptor IFNAR1 on CD8^+^ cytotoxic T lymphocytes (CTLs) (44). These events rely on poly-ADP ribose polymerase-11 (PARP11), which is induced in tumor CTLs and serves as a key regulatory factor for the immunosuppressive TME. Ablation of PARP11 can prevent the loss of IFNAR1, increase the antitumor activity of CTLs, and inhibit tumor growth in an IFNAR1-dependent manner. Therefore, the genetic or pharmacological inactivation of PARP11 increases the therapeutic efficacy of CAR-T cells. CAR-T-cell-based therapy designed to target PARP11 has shown superior efficacy against solid tumors.

### 5.6 Controlling off-target toxicity

To alleviate the “on-target, off-tumor” toxicity of CAR-T-cell therapy, it is necessary to carefully balance effective T-cell activation to ensure antitumor activity and decrease the potential for uncontrolled activation, which may generate immunopathology. The inducible cysteine aspartate protease 9 (iCasp9) “safety switch” provides a solution to remove improperly activated CAR-T cells (171). The induction of iCasp9 depends on the administration of the small-molecule dimerization drug AP1903, which rapidly induces apoptosis in transduced cells and prioritizes the killing of activated cells expressing high levels of transgenic genes. The iCasp9 gene has been incorporated into the vector used for preclinical studies and has demonstrated effective and reliable suicide gene activity in phase 1 clinical trials. By significantly improving the safety of CAR-T-cell therapy, iCasp9/AP1903 suicide gene technology can promote its wider clinical application.

In addition to ensuring the safety of CAR-T-cell therapy, increasing the targeting ability of CAR-T cells is crucial. One of the characteristics of tumor cells is the mixed expression of antigens. Designing dual-targeted CAR-T cells can maximize their ability to prevent tumors from escaping immune system detection and limit their off-target toxicity. Dual-targeted CAR-T-cell therapy can achieve dual effects, selectively targeting another B-cell malignant tumor target while targeting CD19. In this way, even if cancer cells develop an evasion mechanism, this therapy can switch targets in a timely manner to continuously attack cancer cells. Roybal et al. designed a combined and activated T-cell circuit in which a synthesized Notch receptor for one antigen induced the expression of a CAR for a second dual-target antigen. These dual receptors and gate T cells are armed and activated only in the presence of dual-antigen tumor cells. This precise dual receptor circuit opens the door to a wider range of immune recognition for tumors (172). Editing ready-made CD19/CD22 dual-targeted CAR-T cells through CRISPR can allow these edited cells to be used as a new treatment for ALL (173).

If the dose of CAR-T cells can be precisely controlled at different time points, toxic side effects can be reduced. Mestermann et al. used the tyrosine kinase inhibitor (TKI) dasatinib as a lead compound (174). Dasatinib eliminates CAR signaling by blocking the adenosine triphosphate binding site of LCK and immediately blocks the function of CD8^+^ and CD4^+^ CAR-T cells. This blocking effect does not affect the activity of CAR-T cells and is quickly and completely reversible after the removal of dasatinib, confirming that dasatinib can be used as a clinical on/off switch for CAR-T cells. Similarly, the clinically approved drug lenalidomide can also serve as an ON and OFF switch for CAR-T cells (175). Another study identified “super degron” tags that increase sensitivity to lenalidomide-induced degradation and used these degradable tags to generate off-switch-degradable CARs. A lenalidomide-induced dimer system was designed to obtain split CARs that require both lenalidomide and target antibody activation as ON switches. *In vivo*, ON-switch split CARs exhibit lenalidomide-dependent antitumor activity and OFF-switch degradation of inflammatory cytokines while maintaining antitumor efficacy. By utilizing synthesized Notch receptors and transcriptionally connecting multiple molecules to recognize events, different multireceptor cell recognition circuit libraries can be constructed (176). These synthetic circuits allow engineered T cells with integrated extracellular and intracellular antigen recognition, providing robustness to heterogeneity, and enable achieve precise recognition by integrating up to three different antigens with positive or negative logic. Connecting multiple molecular recognition events in synthetic circuits provides a powerful method for designing cell-level recognition.

Abnormal secretion of cytokines is also one of the factors leading to off-target toxicity in CAR-T cells. By designing CAR-T cells that self-regulate the production of inflammatory cytokines, researchers can simultaneously address the toxicity of CRS and increase its ability to attack tumors, which is highly important for the treatment of cancer patients. One study designed CD19 CAR-T cells capable of secreting anti-IL-6Ra single-stranded variable fragment (scFv, known as Toci), which can reduce the severity of CRS (177). In a humanized NSG-SGM3 mouse model, CAR-T cells that secreted single-chain variable fragments (Toci) derived from tocilizumab produced lower CRS-related toxicity, resulting in greater safety than single-dose systemic administration of tocilizumab. Compared with traditional CD19 CAR-T cells, these Toci-secreting CAR-T cells show superior *in vivo* antitumor efficacy. This method of engineering T cells to self-regulate the production of inflammatory cytokines is a clinically compatible strategy that may simultaneously improve safety.

### 5.7 Remodeling the TME

#### 5.7.1 Targeting immunosuppressive cells

In addition to targeting tumor cells directly, targeting other cells in the tumor microenvironment is a promising method. Tumor-associated macrophages (TAMs) are the most widely infiltrating immune cells in the TME. In clinical practice, a high number of TAMs is closely related to a poorer prognosis of various cancers. Because of the crucial role of TAMs in tumor development, the clearance of TAMs may be a method to alter the immunosuppressive environment of the TME and promote an antitumor immune response (178). In a previous study, Sánchez-Paulette et al. designed a CAR-T-cell that targets F4/80 (179), which can not only effectively clear TAMs and relieve the immunosuppression caused by TAMs but also promote the tumor antigen-specific T-cell immune response, thus inhibiting the growth of various tumors. These findings provide support for the subsequent development of CAR-T-cell therapy for clearing TAMs.

#### 5.7.2 Remolding of immunosuppressive cells

Owing to the high plasticity and tumor infiltration ability of macrophages, researchers are attempting to turn them into tumor-killing weapons through gene editing. The “Don’t eat me” signaling pathway involving CD47/SIRPα is a key mechanism by which tumor cells evade macrophage phagocytosis. CRISPR/Cas9 gene editing technology can be used to knock down SIRPα in macrophages, increasing tumor killing and phagocytic ability (180). In 2018, CAR was first used to modify macrophages. Equipping macrophages with tumor antigen-specific CARs creates a potential weapon for targeted tumor killing, especially for solid tumors (181). Cheng et al. designed a series of CARs targeting CD19 or CD22, named CAR Ps. After transfecting these CAR Ps into macrophage cells, they observed strong specificity and phagocytic ability toward human Burkitt lymphoma cells both *in vitro* and *in vivo* (178). Similarly, a 2020 study demonstrated that CAR gene editing in human macrophages can guide their phagocytic activity against tumors (182). Adenovirus vectors can be used to modify HER2 CAR-M cells. Transfecting viral vectors into PBMCs from tumor patients and differentiating them into macrophages,can increase their likelihood of maintaining their tumor-inhibiting activity, which can help them overcome the transition to an immunosuppressive state. One of the main advantages of CAR M-cell therapy is its ability to create a proinflammatory environment within the tumor. The proinflammatory TME is also friendlier to other immune cells, such as T cells. Once T cells enter the tumor, they can recognize tumor antigens presented by macrophages and target cancer cells for destruction. In summary, CAR-M-cell therapy has shown significant advantages in the treatment of solid tumors (183).

The tumor immune response can also be improved by regulating the differentiation of suppressive immune cells. MDSCs are a mixture of immature myeloid cell populations with high heterogeneity and immunosuppressive activity. All-trans retinoic acid (ATRA) can promote the differentiation of MDSCs into granulocytes, macrophages, and DCs and increase the host antitumor immune response by neutralizing the production of ROS (184). The promotion of the development of MDSCs into normal monocytes and granulocytes not only reduces the number of MDSCs but also increases the number of mature myeloid cells, inhibiting tumor growth (185).

### 5.8 Increasing the effectiveness of CAR-T cells by combination therapy

With the emergence and development of various new immunotherapies, combination therapy has gradually become a popular way to treat tumors. The combination of CAR-T cells and immune checkpoint inhibitors may achieve remarkable therapeutic effects (186). PD-1 blockade increases the survival of CAR-T cells and promotes the killing of PD-L1-positive tumor cells. Research data suggest that PD-1-targeted combination therapy may help improve the therapeutic efficacy and persistence of CAR-T cells in patients. The current research further supports the combination of anti-PD-1 monoclonal antibodies and CAR-T-cell therapy and allows its use for treatment in clinical trials of GD2-specific CAR-T cells in patients with neuroblastoma.

When combined with a nanoparticle RNA vaccine (187), the CAR antigen can be delivered throughout the body to the lymphatic regions, stimulating CAR-T cells. The presentation of natural folding targets on resident antigen-presenting cells promotes the homologous and selective expansion of CAR-T cells. This leads to the successful infiltration of CAR-T cells and tumor regression in mouse models. Using CAR-T cells with the help of DC vaccines is a reliable method for solid tumor treatment (188). One study constructed a combined method of CAR-T cells and DC vaccines. After the two types of cells were cocultured, CAR-T cells proliferated extensively and exhibited an increased CD45 RO^+^ CCR7^+^ phenotype. The injection of DC vaccines increased the immune-killing effect of CAR-T cells *in vivo*, the number of CAR-T cells in the peripheral blood of the mice was higher, and they persisted longer, indicating that DC vaccines could increase the persistence of CAR-T cells in mice.

### 5.9 CAR-T-cell recruitment and infiltration

The attraction of chemokines and the interaction between chemokines and receptors are the first steps in the process of T-cell trafficking to tumors. Therefore, modifying CAR-T cells in the tumor microenvironment to target enriched chemokines may be a feasible strategy for optimizing CAR-T-cell trafficking efficiency, especially in solid tumors. One study reported that several CXCR2 ligands are expressed at relatively high levels in human hepatocellular carcinoma tumor tissues and cell lines compared with other chemokines (189). However, both human peripheral T cells and hepatocellular carcinoma tumor-infiltrating T cells lack the expression of CXCR2. In xenograft tumor models, the expression of CXCR2 in CAR-T cells notably promotes tumor infiltration and enhances the antitumor effect of these cells. Another study introduced CXCR2 into CAR-T cells for the treatment of PDAC, and the results revealed that CXCR2 CAR-T cells not only reduced the volume of transplanted PDAC tumors but also completely eliminated the formation of metastatic tumors. CXCR2 plays an important and promising role in increasing the efficiency of CAR-T-cell therapy for primary and metastatic PDAC (190).

By delivering specific antigens to tumor cells in a specific way, we can also increase the specific killing ability of CAR-T cells and improve the infiltration effect. Certain types of bacteria selectively colonize tumor cores with immune privileges and can be engineered into antigen-independent therapeutic delivery platforms. To address the issue of CAR-T-cell infiltration, Vincent et al. developed a probiotic-guided CAR-T-cell (ProCAR) platform (191), in which tumor-colonizing probiotics (*E. coli* Nissle1917) release synthetic targets and label tumor tissue for CAR-mediated *in situ* lysis. This system demonstrated that CAR-T-cell therapy for solid tumors with unknown antigens is safe and effective in various models of human and mouse cancer and is a potential method for treating heterogeneous, cold tumors and poorly infiltrated solid tumors. However, humans are more sensitive than mice to endotoxins. Structural modification of LPS can continuously reduce TLR4 stimulation without disrupting bacterial activity or tumor colonization. This modification further limits bacterial growth and reduces immunogenicity, promoting safe systemic delivery and repeated administration (192).

### 5.10 Utilizing natural mutations in T cells

In human T-lymphomas, the evolution of the disease actively selects for mutations that increase T-cell fitness under challenges similar to those faced by therapeutic T cells. Therefore, it may be possible to use these mutations to improve T-cell therapies. T-cell tumors acquire mutations that increase their adaptive capacity. These mutations can undergo positive selection in the immunosuppressive microenvironment of solid tumors, enhancing the survival and development ability of tumor cells. In a previous study, Garcia et al. constructed a T-cell library containing 71 mutants and 45 wild-type controls (193). These mutations were introduced into human and mouse T cells, and their effects on the T-cell phenotype *in vitro* and *in vivo* were evaluated. This approach identified a novel gene fusion, CARD11-PIK3R3, that is capable of significant T-cell efficacy. CARD11-PIK3R3 promoted CAR-T-cell antitumor activity, reduced T-cell dosage requirements, and alleviated the need for T-cell exhaustion preconditioning. In addition, many researchers have used knockout T lymphoma antioncogenes to enhance T-cell therapy while triggering no malignant mutations (194). However, a patient was reported to develop CAR-T-cell lymphoma after being infused with BCMA-targeted CAR-T cells, which indicated that mutations had occurred before CAR-T-cell manufacturing (195). Therefore, additional safety methods, such as suicide switches, can be used to remodel T cells, increase control over CAR-T cells, and prevent T-cell exhaustion after sufficient treatment efficacy is achieved.

## 6 Conclusion

CAR-T-cell therapy, as one of the most promising methods for cancer treatment, has changed the treatment pattern of some hematologic cancers and may be used for the treatment of solid tumors and other diseases. The high cost of CAR-T-cell therapy is due mainly to its personalization and difficulty in mass production. By constructing cell lines, inducing relevant cells with iPSCs, and performing transient transduction *in vivo*, the production cost of CAR-T cells can be significantly reduced, and their universality can be enhanced. New gene editing technologies, such as the CRISPR/Cas9 system, can increase the specificity of CAR loci and thereby increase the safety of CAR-T-cell therapy. The effective treatment of solid tumors may depend on combination therapy. We can use immunosuppressants, tumor vaccines, and other methods in combination with CAR-T cells and improve the safety of therapy through modification strategies. In clinical trials, the incidence and severity of anti-CAR immune reactions should be reduced, and the immunogenicity should be monitored in patients receiving CAR-T-cell therapy to enable timely and effective responses to possible resistance. To increase the low infiltration rate, we can modify immunosuppressive cells that readily infiltrate the TME so that they express tumor-killing elements instead of inhibitory elements, directly disintegrating the tumor from inside. It is also possible to modify tumor microorganisms to deliver specific antigens to tumor cells and express chemokines. This modification can enhance both antigen specificity and CAR-T-cell infiltration ability. Although many challenges remain in improving the efficacy of CAR-T-cell therapy, especially in solid tumors, and many methods have room for further optimization, we believe that CAR-T-cell therapy is highly promising. The development of CAR-T cells that can be used for the treatment of a wider range of patients can reduce costs and greatly increase the feasibility and popularity of immunotherapy.
